# Therapeutic Application of Anesthesin in Medicine

**Published:** 1905-04

**Authors:** 

**Affiliations:** Practicing Physician at Weistrach


### THERAPEUTIC APPLICATION OF ANESTHESIN IN MEDICINE.*

By DR. HONIGSCHMIED,

Practicing Physician at Weistrach. (From *Die Heilkunde*, VIII, No. 2, February, 1904).

Anesthesin, a paraamidobenzicacidester, is a fine white powder, readily soluble in fatty oils, in ether, and in alcohol, and also in lanolin, wool fat, vaselin, etc. It is scarcely soluble in cold water, and with difficulty only in warm water, in which, however, it is precipitated immediately after the fluid has cooled.

The application, therefore, should be either in the form of a powder, alone, or in combination with other remedies, or in the form of oily or alcohol containing solutions, or in ointments. However, it may be used in mixtures that require to be shaken, or as suppositories and vaginal globules.

In surgery, I have used anesthesin in all instances requiring cauterization, or previous to the union of gaping wounds, or in children, and in very sensitive persons, even without urgent necessity, dusting it with a brush upon wounds, and allowing the dust-

* Translated especially for the Atlanta Journal-Record of Medicine.

like layer to act for about ten minutes before beginning the necessary operation. Cauterization with nitrate of silver and with the Paquelin cautery can be performed without causing any pain whatever. The suturing of wounds was not painful following the application of Anesthesin. As a fundamental principle, we allow sufficient time for the remedy to display its effects; i. e., wait until a portion of the anesthesin is absorbed by the tissues. If pain is present, it will diminish as early as one or two minutes after the application of anesthesin. If the operation is undertaken during the one or two minutes, the application of the remedy will not control pain in the deeper layers.

I was able, after waiting ten minutes, (in children, after a quarter of an hour, for precaution's sake), to make deep cauterizations without the patient's complaining of any pain.

K. B., eight years old, was affected by osteomyelitis of the left tibia. The bone abscess was incised and the cavity scraped and cleaned. The swelling of the bone extended, nevertheless, and sequestra developed at several points. It became necessary to cauterize repeatedly the fungous proliferations, as well as the bone channels. For this reason, after previous cleansing, I dusted the entire infected surface with anesthesin, exercising care that this substance entered the channels, and eventually forced it well into the same by means of a brush, or with a small piece of wood. If this precaution was omitted, cauterization of the bone cavities was painful, and could not be performed with sufficient energy.

J. R., eight years of age, suffered from a transverse cut on the upper arm, about 5 cm. in length. The wound gaped and the edges of the same were 2 cm. apart. I dusted with anesthesin, five minutes later applied a sublimate solution to disinfect the wound, and then waited another ten minutes before suturing. The suturing caused no pain whatever.

In extirpation of the tonsils I attempted to produce anesthesia by dusting with anesthesin; at first I tried to apply the powder with a cotton tampon, but this caused a tickling sensation and nausea. In consequence, I resorted to a blower and dusted not only the tonsils but the entire pharyngeal wall. A sensation of numbness of the parts became manifest after a few minutes. The patients are then more ready to submit to the introduction of the instrument, and it is easier also to adjust the tonsils into the guillotine. This is a great advantage, the extirpation itself requiring but a second; if then the wound surface is touched with anesthesin the burning pain ceases at once and, after cessation of the hemorrhage, we may proceed to remove the second tonsil.

Anesthesia of the deeper tissues does not take place, the anesthesin acting on those places only which are reached by it and at which it is absorbed. While in the case of an open wound resorption took place as a result of long-continued action, rendering the margins of the wound anesthetic, so that the stitches were painless; by the dusting of anesthesin on the tonsils, however, the drug will act merely upon the surface, thereby anesthetizing the latter only.

In painful skin affections and in ulcers it is advisable, before applying the particular remedies, to dust the painful parts very slightly with anesthesin. The effect is remarkable.

One of my patients was a woman forty years old, who suffered from eczema of the face and of the hairy part of the head in which deep ulcers had already formed. I found loretin indicated in this special instance and applied the same. The result was a terribly burning pain which allegedly persisted for the entire day. The next time, I first dusted with anesthesin and then applied a thick layer of loretin. The result was marvelous; there was no sensation of pain whatever.

In cases of itching of the skin it is sufficient to use a ten per cent. ointment for inunction, eventually to prepare a gauze plaster with the ointment and to cover the affected part. I always used:

| | | |
|---|---|---|
| ℞ | Anesthesin "Ritsert," | 10.0 |
| | Vaselin alb. | 20.0 |
| | Alapurin | 70.0 |
| | S. Ointment. | |

This ointment is readily absorbed by the skin, and, according to my experience, gives the most gratifying results.

In ophthalmology I have tried anesthesin for the extraction of a foreign body from the eye. I dusted the lid and waited. Although the pain diminished I did not succeed in rendering the eye anesthetic, and I was obliged to resort to cocainization.

Internally I have employed anesthesin in a great variety of affections.

It promptly relieves pain in diphtheria. When the gangrenous crusts are cast off and the crateriform wound surface of the ulcer becomes visible, the pain is so violent that the little patients refuse to take any nourishment whatever, so that they will not eat, drink, or take medicines. In such cases I have used anesthesin in Mist. oleosa. The pain ceased promptly after the first dose of the remedy, and, as the medicine was given every two hours, the little ones remained free from pain. The dose was 1:100, one "child's spoonful," every two hours.

For the various forms of stomatitis, for instance, stomatitis catarrhalis, aphthous, ulcerous stomatitis, epizootic aphthal, thrush, it is sufficient to dust with anesthesin, or to paint with mucilago anesthesin, made as follows:

℞ Ol. amygdal. dulc.
Spir. vini. rectif.
Mucil. gummi arab.................................. aa 15.0
M. f. emulsio, addae
Anaesth........................................... 1.0
Syr. simpl......................................... 15.0
M. D. S. To paint the affected parts.

With an addition of syrup to the remedy children will gladly take the medicine, or they will readily submit to the painting of the parts.

An addition of menthol is also possible. The usual remedies, such as solutions of potassium chlorate, potassium permanganate, potassium oxymuriate, sodium salicylate, may be employed without giving rise to any perception or manifestation of pain.

In tuberculous laryngitis, I have administered anesthesin in Mist. oleosa. However, I have made experiments also with insufflations, inhalations and painting. The results were uniformly favorable in all cases. That the method of employment changes, follows from the fact that the patient himself desires the employment of something new, hoping that that will result in cure. To many patients, insufflations into the larynx are the least agreeable, as the entrance of the powder into the rima glottidis may sometimes give rise to a temporary irritation and cough, which is never the case in a different form of application.

I have seen good results follow the administration of anesthesin in gastric ulcer, in cancer of the stomach, and in simple hyperesthesia. I gave the remedy in these cases either as powder alone, or in combination with Pulv., rad. Rhe, with bismuth subnitrate, or with alkalies. The single dose was 0.2 to 0.5 (3 to 7½ grs.) three to four times a day.

The following combination gave good results in cases of intestinal inertia:

℞ Anesthesin "Ritsert"....................................3.60
Pulv. rad. Rhei chin.
Magist. Bism. āā........................................0.50
Sacch. lact..............................................2.00
M. f. pulv. div. in dos..................................XII

D. S. One powder three to four times daily.

In carcinoma of the stomach, it is necessary to employ larger doses. In an advanced stage, I prescribe 0.5 (7½ grs.) grams of anesthesin, to be taken four times daily, either in water or in wafers.

In ulcerated carcinoma of the breast or of the uterus, etc., anesthesin alleviates pain immediately, either as a dusting powder or as alapurin ointment.

This is a great acquisition for the patient, as well as for the physician, as formerly it was only possible to keep the patients tolerably free from pain by large doses of narcotics, and, of course, in a constantly dazed condition; whereas, with the employment of this remedy, simple dusting or the application of an ointment is sufficient to relieve the pains for some time.

